# Dyeing of synthetic fiber-based wool blended fabrics in supercritical carbon dioxide

**DOI:** 10.1038/s41598-024-81417-8

**Published:** 2024-12-23

**Authors:** Abdalla A. Mousa, Fatma A. Mohamed, Saadia A. Abd El-Megied, Yehya A. Youssef

**Affiliations:** Dyeing, Printing and Textile Auxiliaries Department, Textile Research and Technology Institute, National Research Centre, 33 EL Buhouth St., Dokki, 12622 Giza Egypt

**Keywords:** VS reactive disperse dye, SC-CO_2_ dyeing, Aqueous dyeing, Wool blend fabric, Polyester fabric, Environmental sciences, Chemistry

## Abstract

Development of supercritical carbon dioxide (SC-CO_2_) dyeing technology for natural fabrics and their blended fabrics is essential for the textile industry due to environmental and economic considerations. Wool (W), polyester (PET) and nylon (N) fabrics and their wool/polyester (W/PET) and wool/nylon (W/N) blended fabrics were dyed in SC-CO_2_ medium with a synthesized reactive disperse dye containing a vinylsulphone (VS) reactive group, which behaves as a disperse dye for synthetic fibers and a reactive dye for protein fibers. The SC-CO_2_ dyeing performance of all fabrics was investigated in terms of color strength, fixation, colorimetric and fastness measurements and compared with the conventional aqueous dyeing method. The results obtained indicate that the VS reactive disperse dye structure and non-polar PET component mainly improved colour strength (K/S) values of the dyed PET fabric and W/PET blended fabrics in SC-CO_2_ compared with those in the aqueous medium. Also, SC-CO_2_ dyeing has a notable influence on a*, b* and C* values of the dyed PET, N and W/PET fabrics and showed that the uptake of the VS reactive disperse dye and their appearance colors are higher and more saturated than the aqueous dyed samples. The levelling and fastness properties of all dyed fabrics in SC-CO_2_ medium are similar to those obtained in the aqueous medium. It was observed that VS reactive disperse dye penetrates well into the PET fabric and is chemically bound with the W fabric using both SC-CO_2_ and aqueous media and did not display significant color difference (∆E) values of W, PET and W/PET fabrics even after 20 washing cycles. The study claims that the VS reactive disperse dye structure and dyed PET-based wool blended fabric are good candidates for industrially SC-CO_2_ dyeing technology.

## Introduction

The fabric blends have continuously grown over recent years. W/PET blend fabric has many superior properties over pure wool fabric for woven apparel goods (e.g. men’s suits and knit dresses). W/N blend fabric is a very popular blend for woven apparel and carpets. It can show complementary properties in terms of crease recovery, durability, abrasion resistance, fast drying, and dimensional stability. Conventional aqueous dyeing methods of blended textiles poses certain obstacles due to the difficulty in selecting compatible dyes and chemicals with better color match, high dyeing performance and the vast color difference of dye up-take on each fiber component. Generally, conventional aqueous dyeing methods of textiles have always involved water intensive processes, with an estimated 100 kg of water required per kg of textiles. Water is a finite resource, and the aqueous dye house effluents contain large amounts of dyes, salts and alkali which are required for the coloration of textiles. Therefore, the importance of eco-friendly dyeing methods of blended textiles is increasing. A non-aqueous dyeing technology using liquid paraffin, siloxane, vegetable oil, etc. as an environmentally friendly dyeing method instead of aqueous dyeing has achieved salt-free dyeing of cotton fibers without discharging wastewater^1–5^. In continuation of the water-free dyeing technologies, SC-CO_2_ is the perfect replacement for the aqueous medium to overcome the problems of conventional dyeing^6–14^. Potential applications of SC-CO_2_ dyeing technology have been efficiently used for dyeing synthetic fibers. Due to the hydrophobic nature of SC-CO_2_ and its non-polarity, synthetic fibers have been successfully dyed with disperse dyes, which have much better solubility in SC-CO_2_ than other classes of dyes. Furthermore, SC-CO_2_ dyeing of PET fabrics as an alternative to aqueous dyeing processes results in environmental advantages, including no fresh water, no wastewater, no auxiliaries, a small amount of hydrophobic disperse dyes, residual hydrophobic disperse dye can be reused and no air pollution as the SC-CO_2_ can be recycled^15–23^. Importantly, DyeCoo Company has developed a patented and industrially proven dyeing technology based on SC-CO_2_, instead of water, to dye PET fabric and yarns^24^. With DyeCoo Company and its 14 industrial machines in Taiwan, Vietnam and Thailand, brands and manufacturers have the opportunity to decrease water and chemical usage significantly, reduce CO_2_ emissions, reduce energy consumption from 20 to 60% and eliminate air pollution as the SC-CO_2_ can be recycled^25^. For further clarification, the industrial DyeCoo SC-CO_2_ dyeing consumes lower energy than industrial aqueous dyeing due to no drying, no steam and shorter dyeing times. DyeCoo reported that the possible range of reduction of CO_2_ emissions from the industrial SC-CO_2_ dyeing process may range from 25 to 40% compared with industrial aqueous dyeing processes. Moreover, there are no other man-made greenhouse gas emissions from the industrial SC-CO_2_ dyeing process like water vapor. Additionally, Yeh Group in Thailand has used DyeCoo’s machines and reported that the industrial DyeCoo SC-CO_2_ dyeing process allowed a 50% reduction in energy^26^. However, SC-CO_2_ dyeing of natural fabrics has not been widely applied because conventional dyes for natural fabric have water-soluble sulfonic acid groups, which cannot be dissolved in the non-polar SC-CO_2_ medium^27,28^. To solve this problem, several reactive disperse dyes have been reported for SC-CO_2_ dyeing of natural fabrics and their blended fabrics including fluorotriazine, chlorotriazine, bromoacrylic acid, halogenated acetamide and VS reactive groups. One major advantage of using reactive disperse dyes for SC-CO_2_ dyeing of natural fabrics that they are non-polar and potentially soluble in non-polar SC-CO_2_^28–42^. Some studies have reported on the dyeing of N fabric with reactive disperse dyes under SC-CO_2_^43,44^.

Many other research activities have applied some temporarily solubilized sulphatoethylsulphone reactive disperse dyes to natural, synthetic and their blended fabrics using conventional aqueous dyeing methods^44–51^. The hydrophobic/hydrophilic nature of wool fabric makes it promising for SC-CO_2_ dyeing compared to the hydrophilic nature of cotton fabric, which makes it challenging. In other words, the hydrophobic surface of wool fiber may improve the affinity and diffusion of the VS reactive disperse dye molecules into wool fibers. It is worth mentioning that the amino groups in wool fiber may also contribute to increasing the forces of interactions between the wool fiber and dye molecules in SC-CO_2_ media. In this context, dyeing of synthetic fiber-based wool blended fabrics in SC-CO_2_ could be accessible when the dyes are able to react with the amino groups in the wool component along with their affinity towards the synthetic fiber component. As the name implies, the target reactive disperse dyes which have reactive disperse dye structure with reactive group might be beneficial for SC-CO_2_ dyeing of synthetic fiber-based wool blended fabrics. In continuation of our previous work^51^, this work provides a simple route to synthesize VS reactive disperse dye, which is also suitable for commercial scale production. The chemistry of the target dye in the present study has been designed to contain β-naphthol as a coupling component to promote the formation of the hydrazo form (intramolecular hydrogen bonding) of the dye chemical structure along with a non-polar VS group, optimizing the dye solubility and uptake in SC-CO_2_. The main objective of this work is to investigate the dyeing behavior of W, N, PET and their PET/W and N/W blended fabrics in SC-CO_2_ using the synthesized VS reactive disperse dye and in this regard the different factors that may affect this process are investigated.

## Materials and methods

### Materials

Four types of mill-scoured and bleached fabrics were supplied by Misr El-Mahala Co. (Egypt). A 100% W fabric, 210 g/m^2^, had a twill weave (2/2) of equal warp and weft (26 × 24 threads/cm, yarn count Nm 44/2). A 100% PET fabric, 149 g/m^2^ had a continuous filament plain weave of warp and weft (72 × 32 threads/cm, yarn count tex 150 × 1). The W/PET blend fabric (55/45), 165 g/m^2^, had a plain weave of warp and weft (21 × 19 thread/cm, yarn count Nm 42/2). The W/N blend fabric (70/30), 165 g/m^2^, had a plain weave of warp and weft (32 × 32 thread/cm, warp yarn count Nm 36/2, weft yarn count Nm 18/1). Also, mill-scoured and bleached single jersey knitted N fabric, 114 g/m^2^, 135d/30f was obtained from El-Shourbagy Co. (Egypt). All the fabrics were treated in an aqueous solution containing 2 g/l nonionic detergent (Sera Wash M-SF, DyStar, Egypt) for 1 h at 80 °C and at a 50:1 liquor ratio, then washed thoroughly with water to ensure the removal of any undesirable contaminants, such as oils that may exist in all types of fabrics and air-dried at room temperature. 1-aminobenzene-4-β-sulphatoethylsulphone was obtained from Amar Impex, India. Other chemicals used in the study were of laboratory reagent grade.

## Methods

### Preparation of model VS reactive disperse dye

**Fig. 1:**
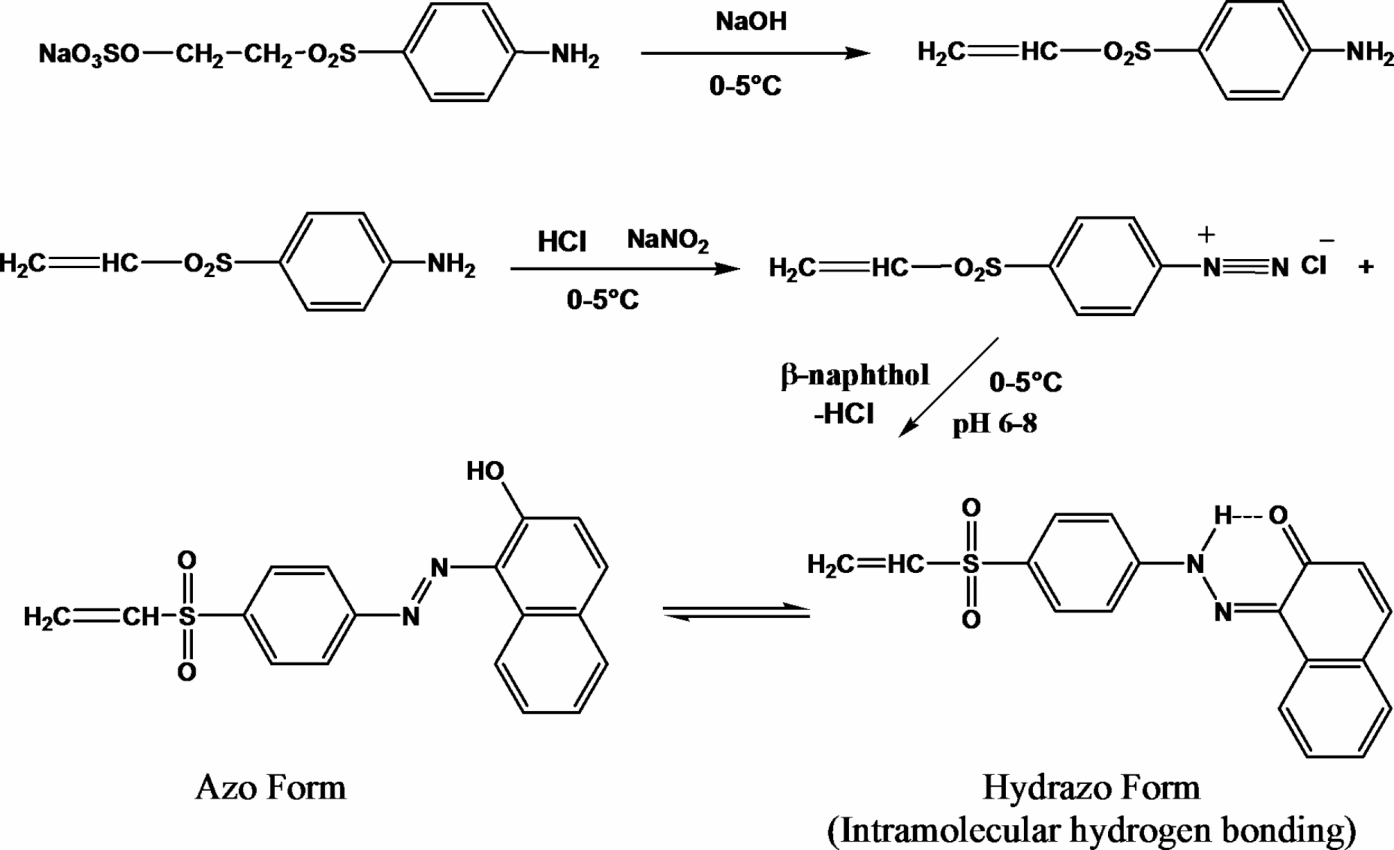
Synthesis of 2-Hydroxy-1-((4-vinylsulfonyl) phenyl) naphthalene VS dye.

The reactive disperse dye, 2-Hydroxy-1-((4-vinylsulfonyl) phenyl) naphthalene, of VS dye structure was synthesized using a similar procedure previously described in literature^44^. As presented in Fig. 1, 1-aminobenzene-4-β-sulphato-ethylsulphone was dissolved in water at room temperature and treated with sodium hydroxide solution at 0–5 °C to form the vinylsulphone analogue, which was diazotized and coupled with β-naphthol in alkaline condition according to the usual procedure. A percent yield of 84% was obtained. The prepared VS dye was used in SC-CO_2_ and aqueous dyeing of W, PET and N fabrics and their W/PET and W/N blend fabrics.

### SC-CO2 dyeing

**Fig. 2:**
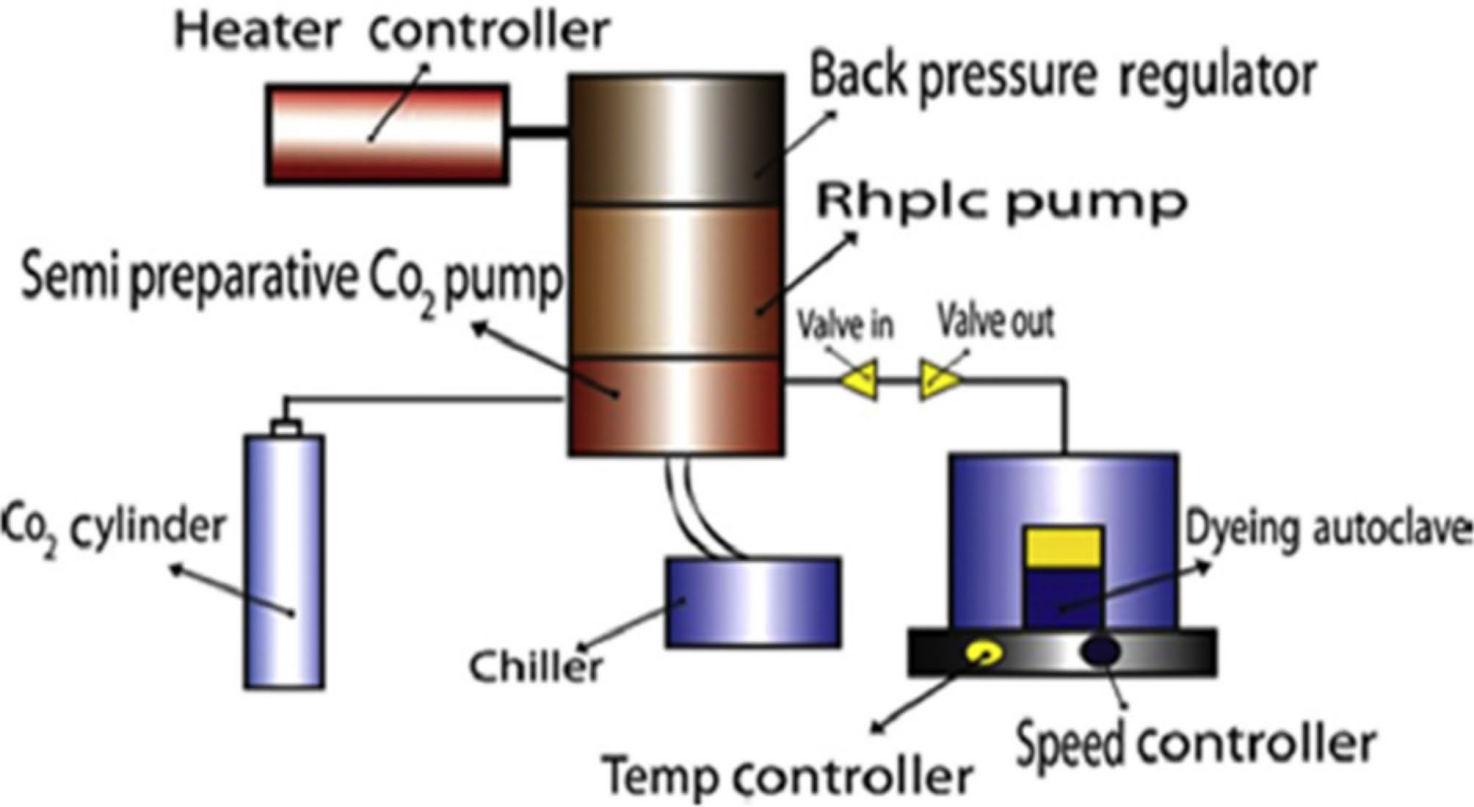
A diagram of the lab-scale SC-CO_2_ dyeing apparatus.

The SC-CO_2_ apparatus, as shown in Fig. 2, consists of the main parts: a cylinder of carbon dioxide, chiller (model Julabo FL601), a semi-preparative CO_2_ pump (model JASCOPU-4386), RHPLC pump for extraction (model JASCO PU-4180), a back pressure regulator (model JASCO BP-4340) with a maximum pressure rate of 300 bar, a heater controller (model HC-2068-01), a dyeing autoclave, a temperature controller and a speed controller (model EYELARCX-1000 H) with a maximum temperature rate of 130 °C, and an internal capacity of approximately 50 ml. The maximum CO_2_ flow rate of the circulation pump is 10 mL/min, but it can reach 30 ml/min with an optional heater^42^.

The appropriate amount of VS dye (2% owf shade) was placed inside the SC-CO_2_ dyeing vessel without a dispersing agent. The W, PET and N fabrics, and blends of W/PET and W/N fabrics were added into the SC-CO_2_ dyeing vessel. The CO_2_ fluid preheated, and then charged into the dyeing vessel. The dyeing was continued at both temperature and pressure required for 1 h. After the dyeing cycle is completed, the machine automatically cooled down to a safe temperature and pressure before the vessels can be opened and the dyed fabrics were then soaped to remove the surface dye, rinsed thoroughly for 2 min with cold water and air-dried for colour measurements and color fastness.

### Aqueous dyeing

**Fig. 3:**
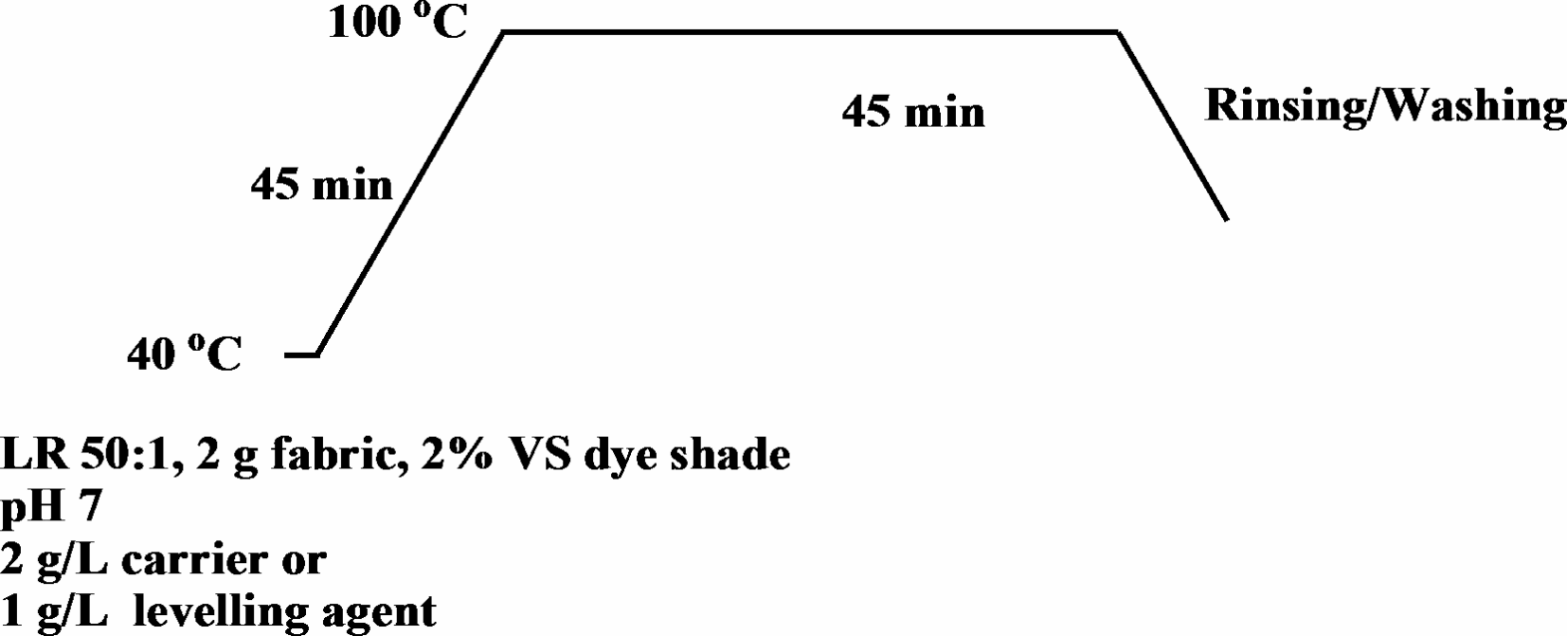
Lab-scale aqueous dyeing curve.

The same amount of VS dye (2% owf shade) was initially premixed with an appropriate amount of nonionic dispersing agent (Sera Gal P-LP; DyStar, Egypt) in a ratio of 1:1, which was then added with stirring to the dye bath at a liquor ratio of 50:1. In the case of the PET and W/PET, the dye bath pH was adjusted to 7 in the presence of 2 g/L low-odour carrier (Sera Gal P-EW; DyStar, Egypt). For the W, N and W/N fabrics, the dye bath pH was adjusted to 7 and the addition of 1 g/L levelling agent (Lyogen NH liquid; Clariant, Egypt). All fabrics were dyed in a Pyrotec infrared laboratory dyeing unit (Roaches International) using 2 g samples^51^. Dyeing started at 40 °C and the temperature was then raised to 100 °C over 45 min. Dyeing continued at the desired temperatures for a further 45 min (90 min. total dyeing time). After dyeing, the PET dyed samples were removed, rinsed in warm water, and subjected to reduction clearing in a solution comprising 2 g /L of sodium hydrosulphite and 2 g /L of sodium hydroxide for 10 min at 60 °C, with a liquor ratio of 1:40. The reduction-cleared sample was then rinsed thoroughly in water. All the dyed samples were soaped with 2 g/l nonionic detergent (Sera Wash M-SF, DyStar, Egypt) for 30 min at 60 °C and at a 50:1 liquor ratio, then thoroughly rinsed in water and air-dried for color measurements and color fastness evaluation (Fig. 3).

#### Color measurements

The K/S values and color coordinates (L*, a*, b*, C* and *h*°) of the dyed W, PET and N fabrics and their W/PET and W/N blend fabrics were measured using an UltraScan PRO spectrophotometer (Hunter Lab, USA). L* represents the lightness coordinate, a* represents the redness/greenness coordinate, with + a* indicating red, and -a* indicating green, b* represents the yellowness/blueness coordinate, with + b* indicating yellow, and -b* indicating blue, C* represents the chroma coordinate and *h*° represents the hue angle, expressed in degrees, with 0° being a location on the + a* axis (red), continuing to 90° for the + b* axis (yellow), 180° for - a* (green), 270° for - b* (blue), and back to 360° = 0°. The levelling of the all dyed fabrics in SC-CO_2_ and aqueous medium was evaluated by measuring the ∆E within each fabric sample at five separate points and the average ∆E between these points was determined^52,53^.

The fixation value of VS dye (%F), which is the percentage of the dye fixed on W and N fabrics and their W/N blend fabric in SC-CO_2_ and aqueous medium relative to the exhausted dye, was measured by refluxing the dyed fabrics in 50% aqueous dimethylformamide (DMF) for 15 min (20:1 liquor ratio) to extract the unfixed dye^51^ using Eq. (1).

1 $$\:\%F=\:\frac{{(K/S)}_{2}}{{(K/S)}_{1}}\:\times\:100$$

where (K/S)_1_ and (K/S)_2_ are the color strength of the dyed samples before and after extraction, respectively.

#### Color fastness testing

The color fastness of the SC-CO_2_ and aqueous dyed W, PET and N fabrics and W/PET and W/N blended fabrics were assessed according to ISO standard methods^54–57^. Fastness to washing was carried out according to ISO 105-C06 B2S. The dyed W, PET and W/PET samples were also subjected to reapt washing cycles (5, 10 and 20) to determine the durability color against successive laundering by evaluating the color difference (ΔE) values relative to the samples with zero washing cycle. Fastness to an acidic and alkaline perspiration was carried out according to ISO 105-E04. The colour changing of PP fabrics and colour staining of the adjacent multi-fiber were then assessed with the ISO grey scales. Light fastness was also assessed according to ISO105-B02 using a Xenon arc lamp test.

## Results and discussion

### Characterization of VS dye

**Fig. 4:**
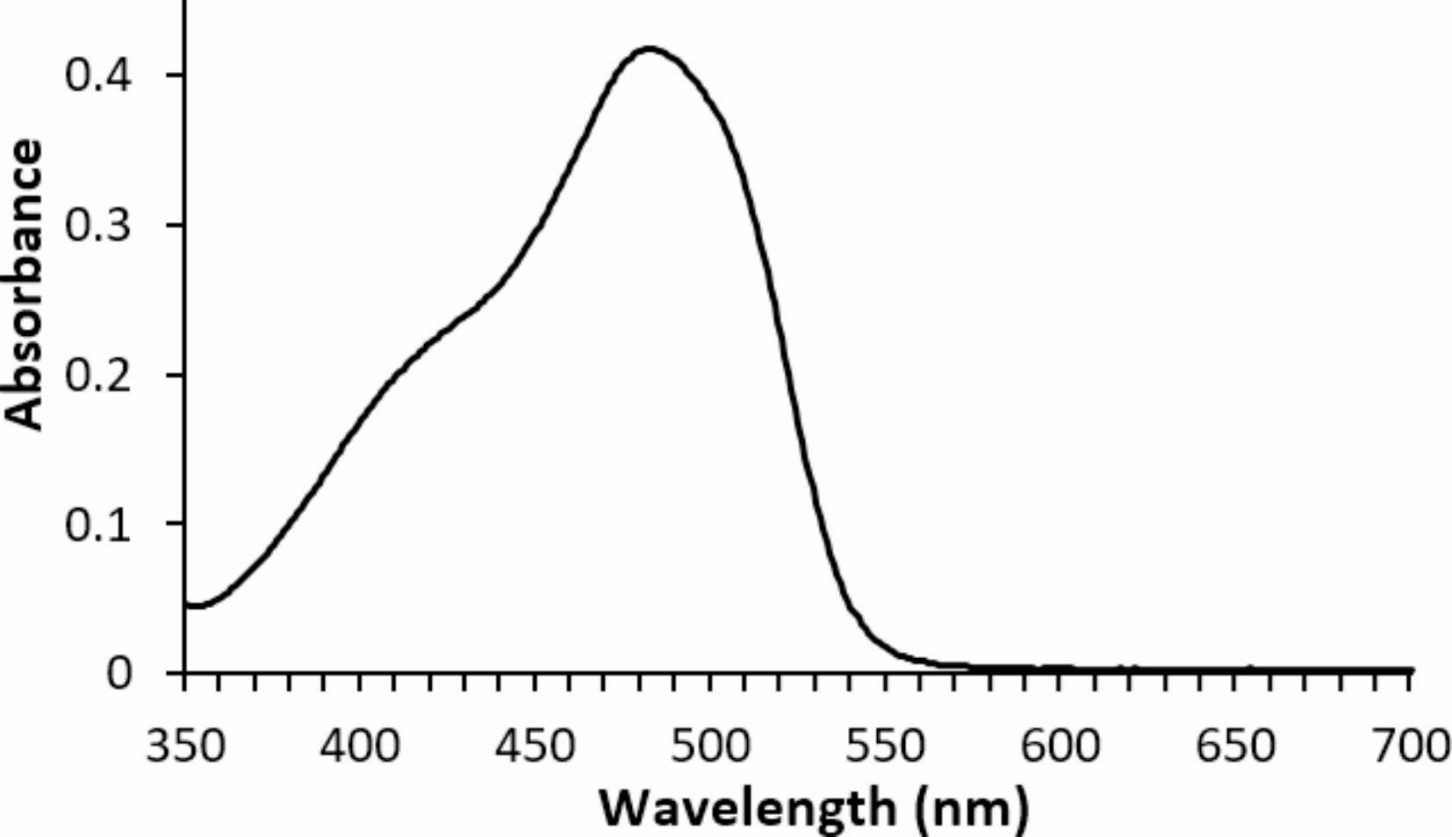
UV-Vs spectrum of VS dye at concentration 2.5 × 10^− 5^ M/L in 50% aqueous DMF solution.

The UV-Vis absorption spectrum (Fig. 4) of the synthesized VS dye, recorded in a 50/50 w/w aqueous DMF solution, showed a maximum absorption peak at 483 nm and the molar excitation coefficient of 2.5 × 10^− 5^ M/L dye concentration was 16,940 L mol^− 1^ cm^− 1^.

**Fig. 5:**
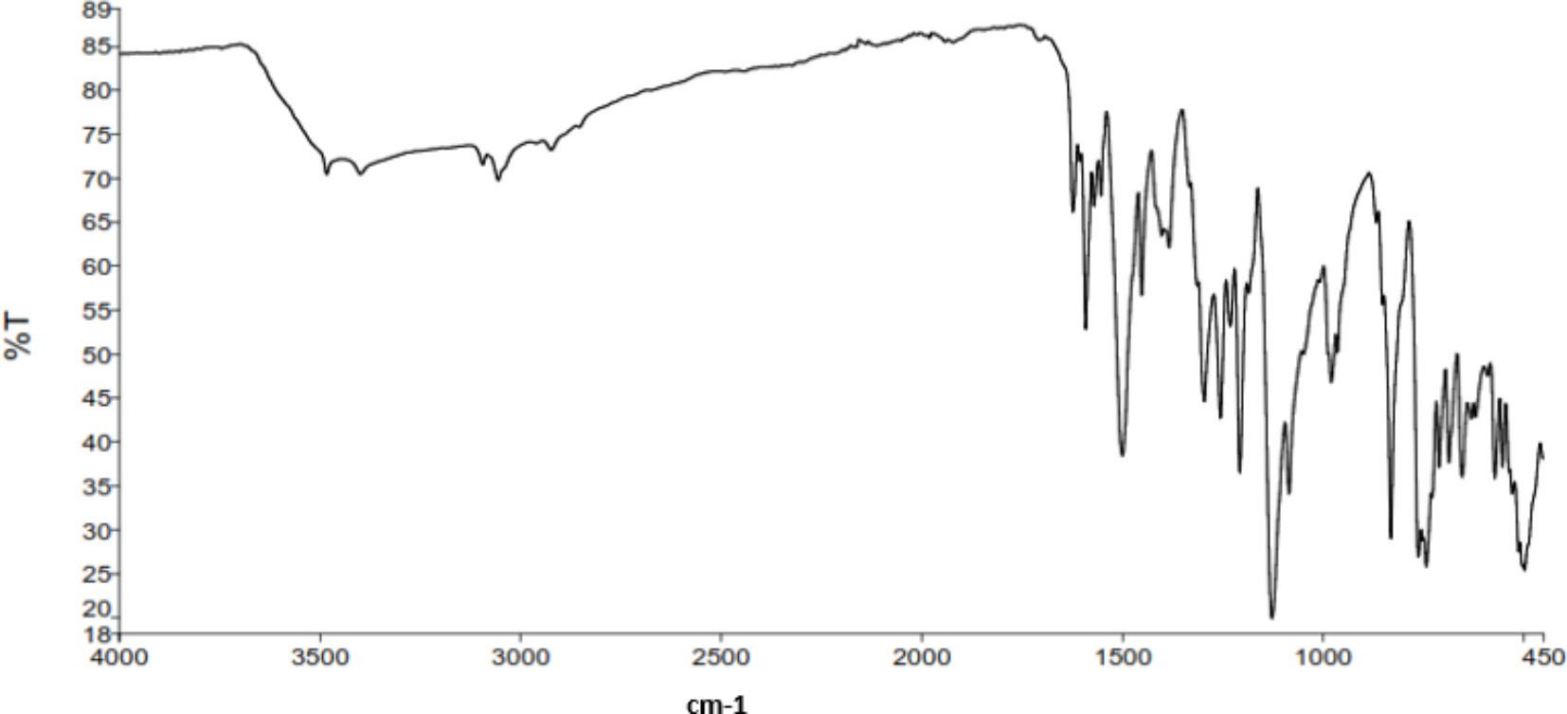
FTIR spectrum of VS dye.

**Fig. 6:**
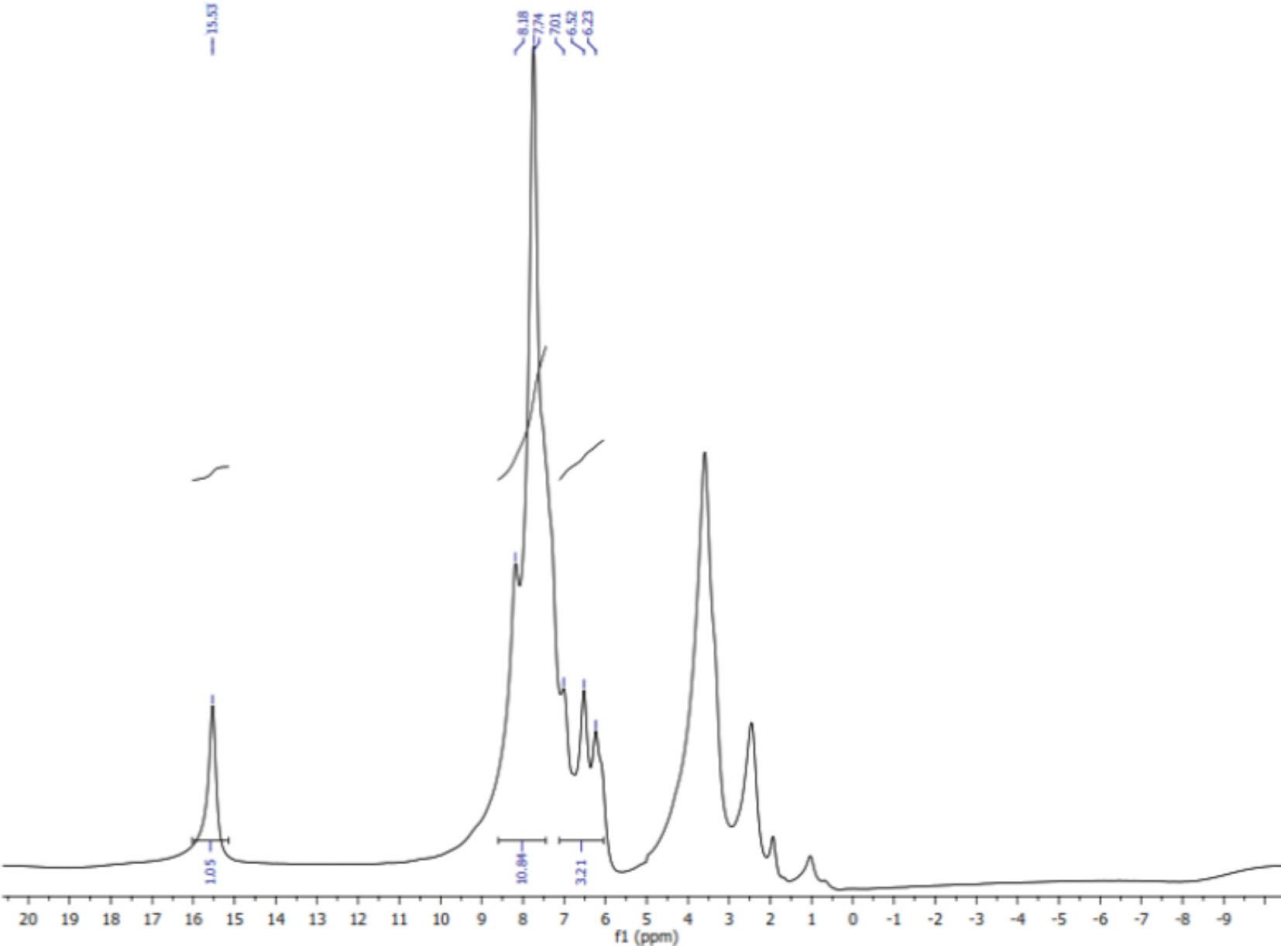
^1^H-NMR spectrum of VS dye.

The IR spectrum of the synthesized VS dye (Fig. 5) showed an absorption band at 3485 –3401 cm^− 1^, corresponding to the characteristic N-H group. The bands at 3057 and 2970 cm-1 were assigned to the aromatic (= C–H) and aliphatic C-H groups, respectively. Also, the appearance of strong peaks at 1591 and 1499 revealed to the existence of = N-NH and –C = N groups, indicating to the hydrazo form. The characteristic absorption band corresponding to the SO_2_ group appeared at 1126 cm^− 1^. The ^1^H-NMR spectrum of VS dye (Fig. 6) was characterized by two peaks at 6.23 and 6.52 ppm, assignable to the protons of the VS group, respectively. The spectrum exhibited multiplet peaks at 7.1–8.18 ppm, integrated to 10 protons assignable to the aromatic naphthyl and phenyl protons. The band at 15.53 ppm was observed corresponding to the hydrazone NH proton.

### Effect of SC-CO2 dyeing temperature

**Fig. 7:**
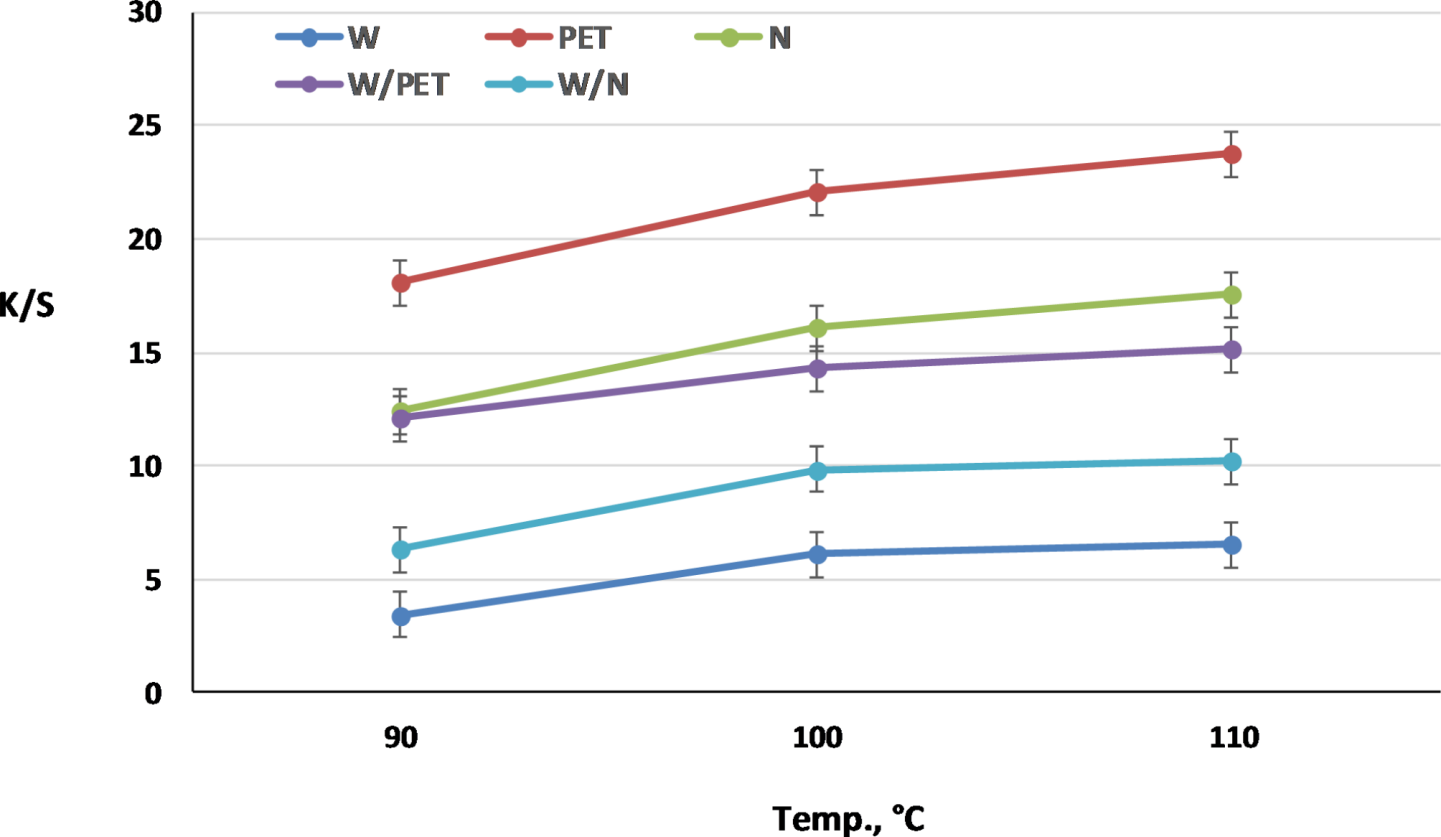
Effect of SC-CO_2_ dyeing temperature on K/S values of W, PET, N, W/PET and W/N blended fabrics at 250 bar, 1 h and 2% shade of VS dye.

Pressure and temperature are two important factors that determine dye solubility in SC-CO_2_. An investigation of the effect of dyeing temperature on K/S values of W, PET and N fabrics and their W/PET and W/N blended fabrics dyed in SC-CO_2_ was performed with the VS dye at a concentration of 2% owf and at constant pressure of 250 bars as shown in Fig. 7. It is clear that increasing the temperature from 90 to 100 °C results in an improvement in the K/S values of all the dyed fabrics. The increase in K/S values is a direct consequence of increasing the VS dye solubility and fiber swelling, thereby enhancing the VS dye diffusion into the fabrics, as more pores and channels are formed in the interior of the fabrics. However, a further increase in SC-CO_2_ dyeing temperature, i.e., beyond 100 °C, has practically a marginal increasing effect on the K/S values of all fabrics. Accordingly, the optimum temperature condition was tailored to the wool fabric types at a relatively mild and promising dyeing temperature (100 °C). The results also reveal that the K/S values of the blends dyeing are mainly determined by the polarity and crystallinity of the fiber components. These findings were further studied to investigate the effect of SC-CO_2_ pressure on the dyeing performance of all fabrics with VS dye at 100 °C.

### Effect of SC-CO2 dyeing pressure

**Fig. 8:**
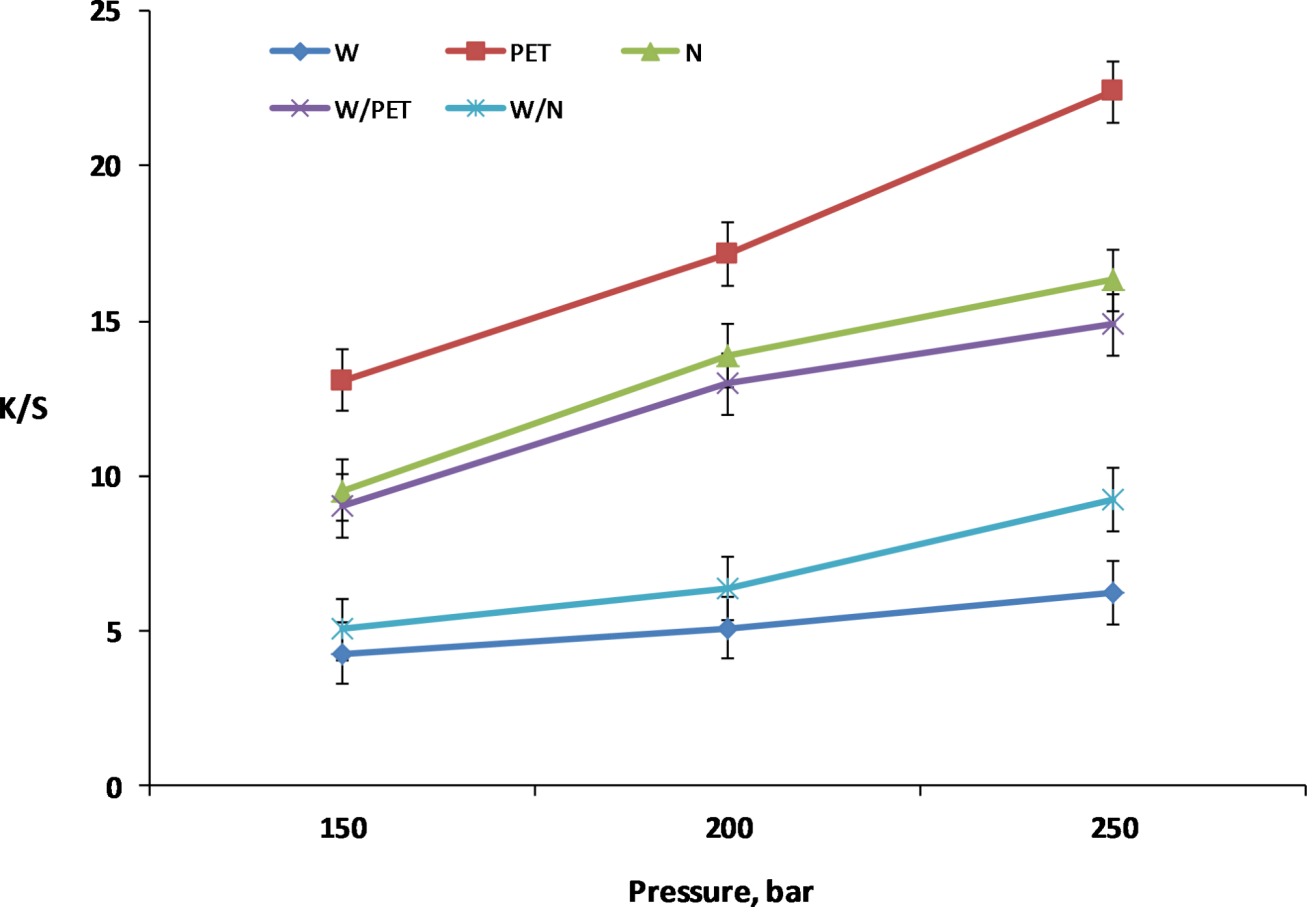
Effect of SC-CO_2_ dyeing pressure on K/S values of W, PET, N, W/PET and W/N blended fabrics at 100 °C, 1 h and 2% shade of VS dye.

The effect of SC-CO_2_ dyeing pressure on the dyeing performance of W, PET and N fabrics as well as their W/PET and W/N blended fabrics with the VS dye was investigated at pressures ranging from 150 to 250 bar at a constant temperature of 100 °C and 2% owf dye concentration, as shown in Fig. 8. It is clear that the K/S values of all dyed fabrics were significantly and linearly improved with system pressures of 150–250 bar. The density of the SC-CO_2_ fluid increases with an increase in pressure, resulting in an enhancement of the VS dye solubility and promoting more interactions between non-polar SC-CO_2_ medium and the intermolecular chains of the fabrics under investigation. Consequently, the adsorption opportunities between VS dye molecules and the fabrics are increased, leading to higher K/S values.

Furthermore, it is clear from both Figs. 7 and 8 that, the K/S values of dyed non-polar PET fabrics at all temperatures and pressures were higher than those of the other dyed fabrics. This is because the VS dye solubility and its affinity to PET fabric become larger, which is in agreement with the dyeing behavior of PET fabrics with disperse dyes in SC-CO_2_^16,58,59^. Theoretically, as shown in scheme 1, the VS dye molecule was designed with a β-naphthol as coupling component, which can predominate the hydrazo form (intramolecular hydrogen bonding) of the dye chemical structure^60^. This resulting in no dye-dye interaction taking place in the SC-CO_2_ medium and consequently can significantly improve the VS dye solubility in the SC-CO_2_ medium. The VS dye behaves as a disperse dye for SC-CO_2_ dyeing of PET fabric and polyester component in W/PET blended fabric as well as a reactive dye for SC-CO_2_ dyeing of W, N and their W/N blended fabrics. Among the dyed fabrics in SC-CO_2_, the order of K/S values found was: PET > N > W/PET > W/N > W. The lower K/S values of dyed N fabrics compared with dyed PET fabrics are attributed to the higher degree of crystallinity of N fabric^61^. The K/S values of dyed W/PET blend fabrics were found to be between those of PET fabrics and those of W fabrics. Also, the K/S values of dyed W/N blend fabrics were found to be between those of N fabrics and those of W fabrics. PET SC-CO_2_ dyeings were excellent due to the non-polarity of PET fabrics that predominates a variety of intermolecular forces of interactions with the VS dye structure; however, the lowest K/S values assigned to W fabric can be attributed to the lower diffusion and interactions of VS dye with the polar W fabric, although the nucleophilic addition reaction between the VS reactive group of the dye molecules and nucleophilic amino in W is possible under SC-CO_2_ conditions. Therefore, the effect of the color range of reactive disperse dye structures incorporating different reactive groups and the blend ratios is a crucial point in SC-CO_2_ blends dyeing and further work will merit investigation.

### K/S values of SC-CO2 and aqueous dyeings

**Fig. 9:**
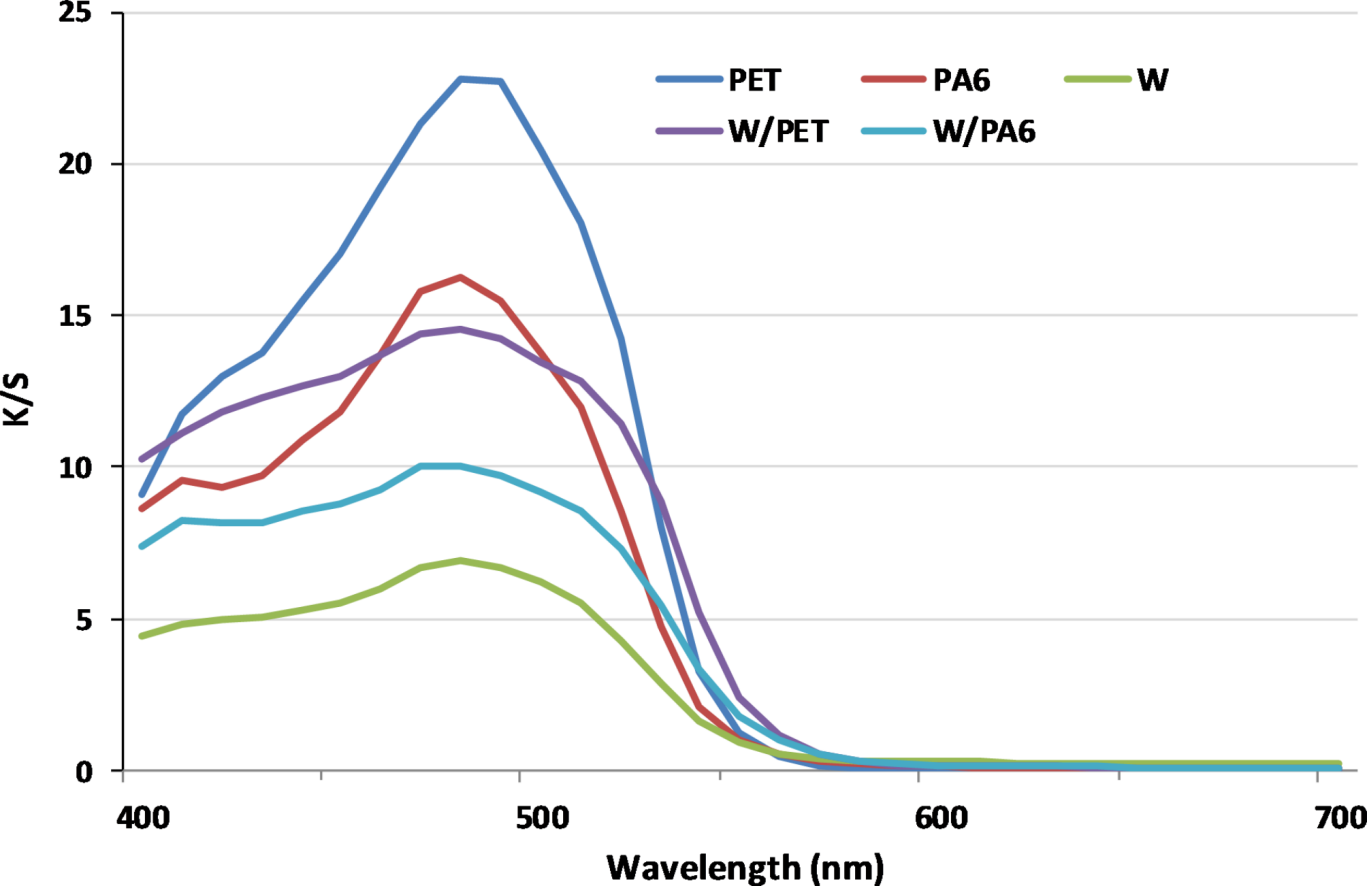
K/S (400–700 nm) values of the W, PET, N and blends of W/PET and W/N fabrics dyed in SC-CO_2_ medium.

**Fig. 10:**
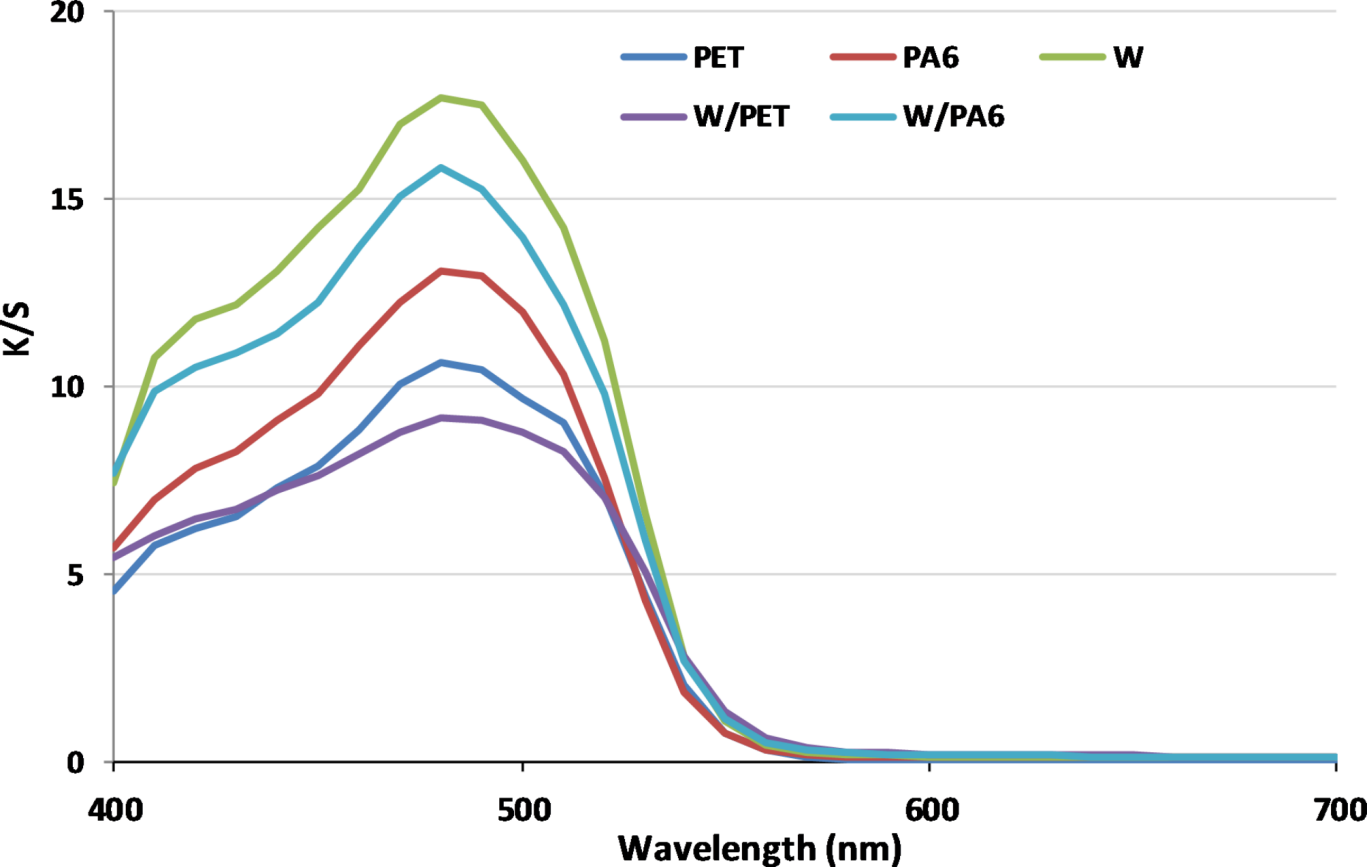
K/S (400–700 nm) values of the W, PET, N and blends of W/PET and W/N fabrics dyed in aqueous medium.

K/S (400–700 nm) values of the dyed W, PET and N fabrics and their W/PET and W/N blended fabrics in SCCO_2_ at 250 bar, 100 °C, 2% shade and 1 h were compared with those in aqueous dyeing at 2% shade, 100 °C, pH 7, and 1 h, as reported in Figs. 9 and 10, respectively.

**Fig. 11:**
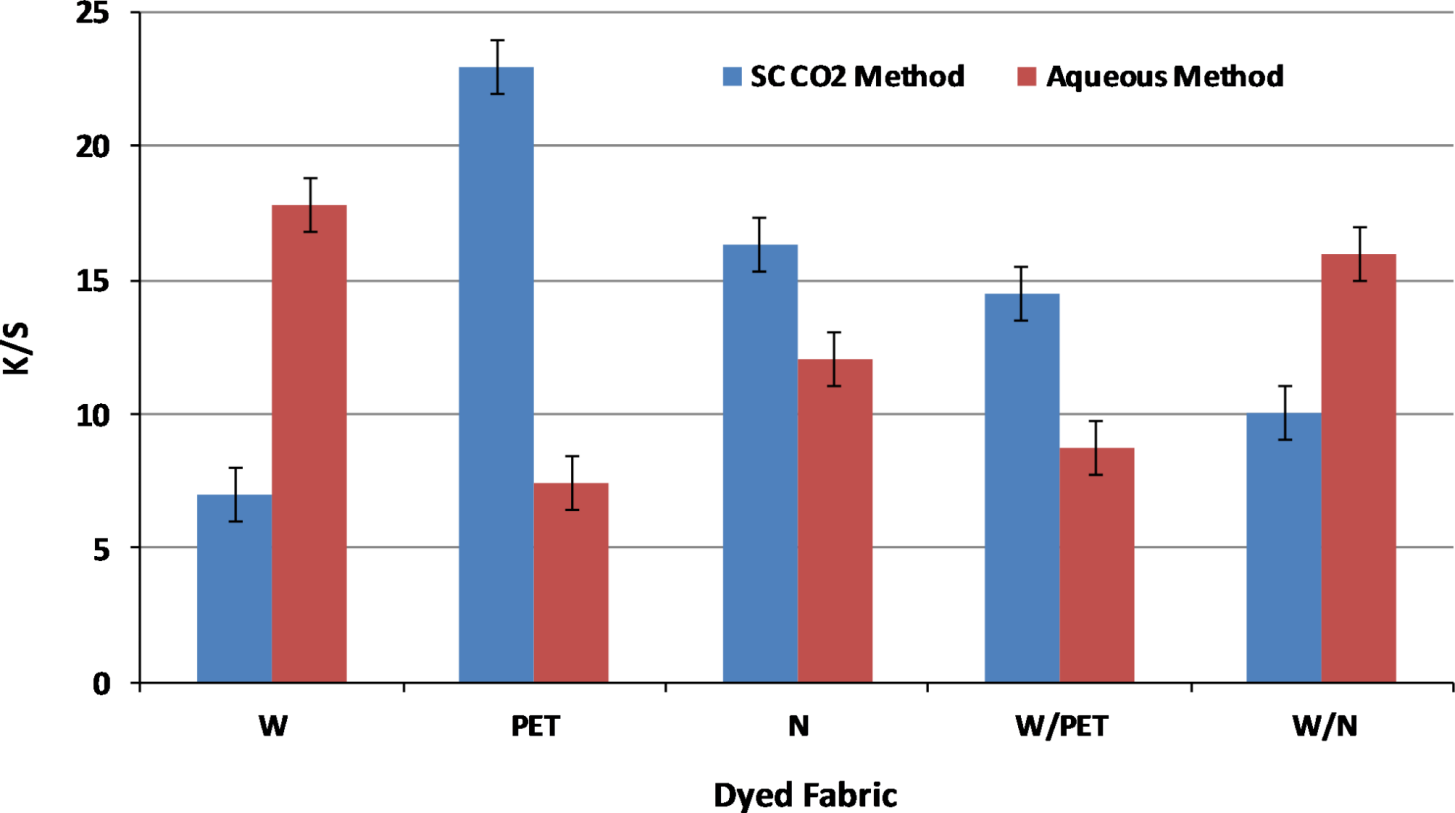
Comparison of K/S values of W, PET, N, W/PET and W/N blended fabrics dyed in SC-CO_2_ medium at (250 bar, 100 °C, 2% shade of VS dye and1 h) and in aqueous medium at (2% shade of VS dye, 100 °C and 1 h).

The comparative results of the dyed PET and W/PET blended fabrics in SC-CO_2_ medium showed higher K/S values than those dyed in aqueous medium, as shown in Fig. 11. This is mainly due to the effect of both the non-polar PET component in W/PET blended fabrics and the high VS dye solubility. Additionally, SC-CO_2_ is known to reduce the glass transition temperature Tg of many polymers considerably, which induces a major beneficial role in increasing the dye transfer inside the polymeric matrix^62,63^. This is also in agreement with that the diffusivity of PET dyeing in the presence of SC-CO_2_ is approximately three orders of magnitude larger than that in aqueous dyeing^64,65^. However, the high ratio of polar W component in W/N blended fabrics resulted in relatively lower K/S values in SC-CO_2_ medium than those dyed in aqueous medium. This is attributed to the fact that, the interactions of the polar W fabric with VS dye are very low in the non-polar SC-CO_2_ medium. Furthermore, the forces of interactions between the VS dye and W component in W/N blended fabrics in the aqueous dyeing are expected to be more than the only chemical bonding of W amino groups and the dye VS reactive groups.

**Fig. 12:**
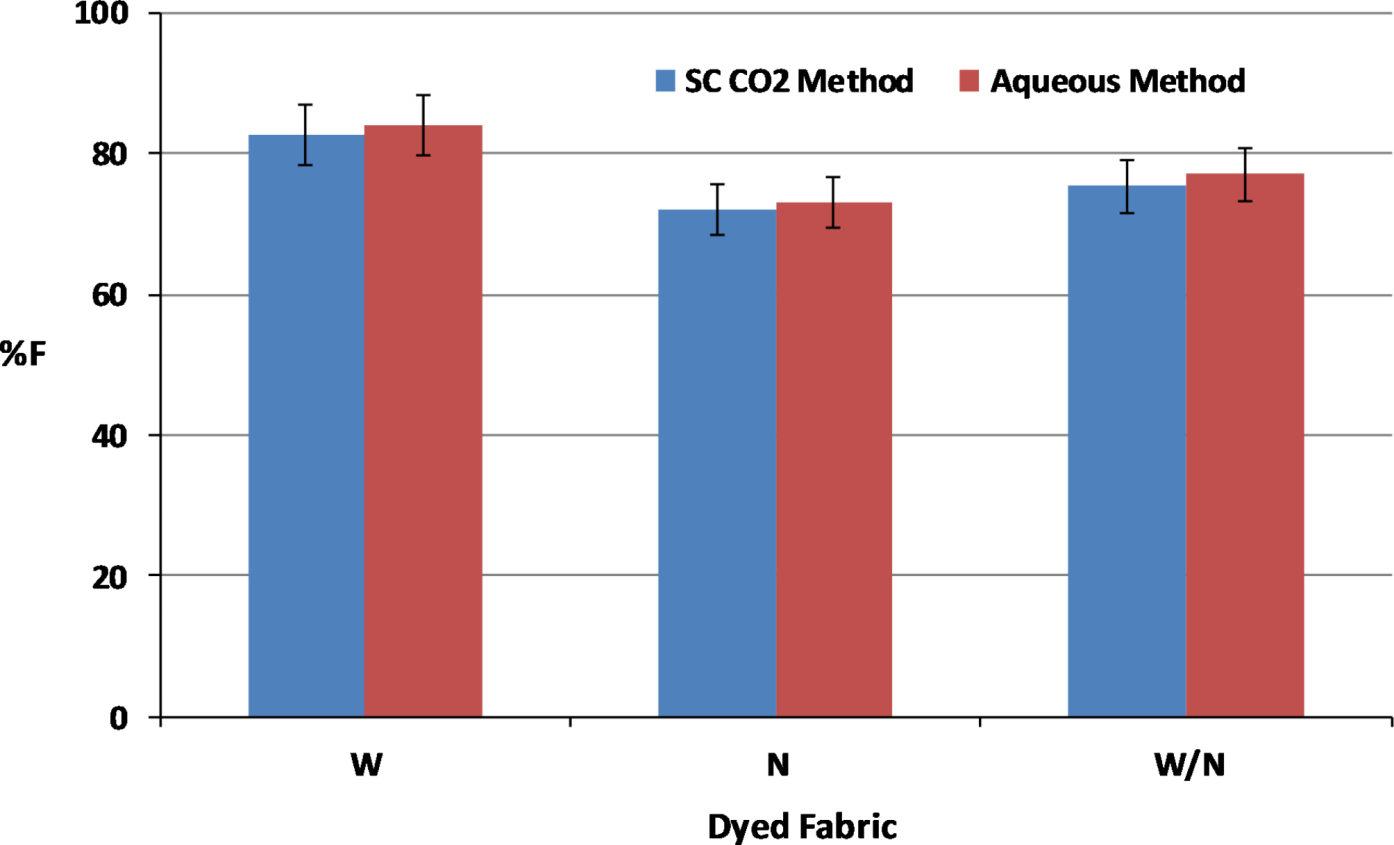
Comparison of dye fixation values of W, N, and W/N blended fabrics dyed in SC-CO_2_ medium at (250 bar, 100 °C, 2% shade of VS dye and1 h) and in aqueous medium at (2% shade of VS dye, 100 °C and 1 h).

**Fig. 13:**
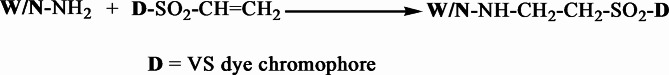
Nucleophilic addition mechanism of VS dye molecule on W and N fabrics under both SC-CO_2_ and aqueous conditions.

Generally, the dye-fibre interactions between VS reactive disperse dye and W/N components in SC-CO₂ or aqueous dyeing medium may involve different mechanisms due to the unique properties of the VS dye structure. In an aqueous medium, water is a polar solvent, facilitating strong hydrogen bonding, Van der Waals and ionic interactions. Also, the W/N functional amino groups can easily be chemically bounded with the VS groups of the dye, allowing for effective dye-fiber covalent bonding. While in SC-CO_2_, the non-polar nature of CO₂ may reduce the ionic interactions. However, the VS groups still react with the nucleophilic sites on the W and N fibers, but potentially at different rates and efficiencies. Bearing this in mind, both the SC-CO_2_ and aqueous dyed W, N and W/N blended fabrics were subjected to a DMF extraction to ascertain the chemical bonding between the VS dye molecules and the amino groups of the fabrics. The VS dye exhibited high fixation on W fabrics with values of 83% and 84% while those of N fabric show fixation values of 72% and 73%, in both SC-CO_2_ and aqueous mediums, respectively, as shown in Fig. 12. The fixation values of the dyed W/N blend fabrics were found to be between those on W fabrics and those on N fabrics, inferring that the VS dye secured relatively high fixation values with the amino-based W and N fibres. Theoretically, there is a nucleophilic addition reaction between the VS reactive group of VS dye molecules and the nucleophilic amino groups in W and N fibers; this reaction is responsible for the fixation of VS dye molecules onto the W and N fibers during the dyeing process in both SC-CO_2_ and aqueous media, as depicted in Fig. 13.

### Color coordinates of SC-CO2 and aqueous dyeings

Table 1 shows the values obtained for the color coordinates (L*, a*, b*, C* and *h*°) of dyed W, PET, N, W/PET and W/N blended fabrics in SC-CO_2_ medium at (250 bar, 100 °C, 2% shade and 1 h) and aqueous medium at (2% shade, 100ºC and 1 h) with VS dye. The lower value of L* represents the darker hue and the higher value refers to the lighter hue of a dyed fabric. The value a* denotes the redness/greenness quality of the dyed fabric with the positive value of a* indicating redness and the negative value of a* indicating greenness. The value b* represents the yellowness/blueness quality of the dyed fabric with the positive value of b* indicating the yellowness and the negative value of b* denoting blueness. The value C* indicates the chroma and the degree of saturation of the color and the value *h*° represents the hue angle, expressed in degrees, with 0° being a location on the + a* axis (red), continuing to 90° for the + b* axis (yellow), 180° for - a* (green), 270° for - b* (blue), and back to 360° = 0°.

**Fig. 14:**
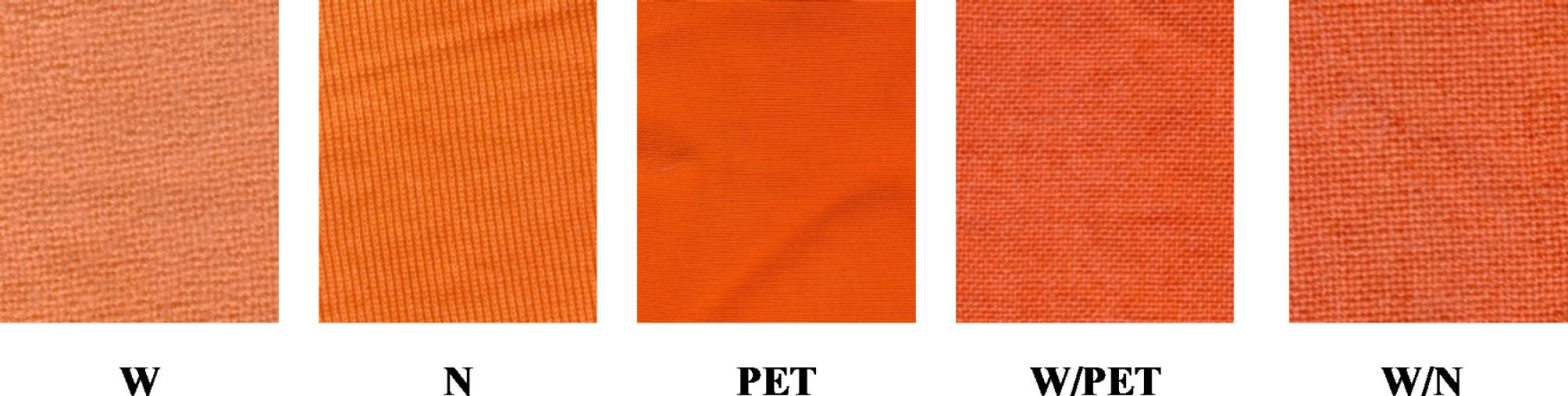
Images of SC-CO_2_ dyed W, N and PET fabrics and W/PET and W/N blended fabrics at (250 bar, 100 °C, 2% shade of VS dye).

It is clear from Table 1 that the dyed PET, N and W/PET fabrics in SC-CO_2_ exhibited darker colors than those dyed in the aqueous medium, as evidenced by the lower values of L*. However, the high L* value of SC-CO_2_ dyed W fabric indicate its lighter colors compared to that in the aqueous medium. The values of b* > a* and both positive in all SC-CO_2_ and aqueous dyed fabrics as well as their *h*° values range from 53.80 to 63.79, indicating the appearance of the orange color of the dye used. The SC-CO_2_ dyeing has a notable influence on a*, b* and C* values of the dyed PET, N and W/PET fabrics and showed that their appearance colors are higher and more saturated than the aqueous dyed samples. Alternatively, the aqueous dyeing of W and W/N fabrics showed higher values of a*, b* and C* than those of SC-CO_2_ dyeing, depending on the higher uptake of the VS reactive disperse dye in the aqueous dyeing. All the dyed W, PET and N fabrics and W/PET and W/N blended fabrics show very good levelling properties, indicating that the SC-CO_2_ dyeing method has a substantially better effect on the ∆E values of the dyed samples, as shown in Table 1. Images of all SC-CO_2_ dyed fabrics are illustrated in Fig. 14.

**Table 1:** Color data of W, N and PET fabrics and W/PET and W/N blended fabrics dyed in SC-CO_2_ and aqueous media.

Dyed samples	Dyeing method	L*	a*	b*	C*	h°	∆E
W	SC-CO_2_	69.64	23.24	42.07	48.06	61.09	0.6 ± 0.1
	Aqueous	61.29	41.54	73.26	84.21	60.45	1.3 ± 0.1
PET	SC-CO_2_	62.81	41.05	74.09	84.71	61.01	0.3 ± 0.1
	Aqueous	67.43	32.40	58.77	67.11	61.13	0.9 ± 0.1
N	SC-CO_2_	61.83	30.90	62.78	69.97	63.79	0.4 ± 0.1
	Aqueous	54.02	26.49	52.28	58.61	63.13	0.6 ± 0.1
W/PET	SC-CO_2_	62.04	37.09	54.76	66.14	55.89	0.4 ± 0.1
	Aqueous	63.19	33.08	54.37	63.65	58.68	0.7 ± 0.1
W/N	SC-CO_2_	61.03	35.35	48.30	59.85	53.80	0.8 ± 0.1
	Aqueous	61.21	38.40	55.64	67.60	55.39	1.1 ± 0.1

**Fig. 15:**
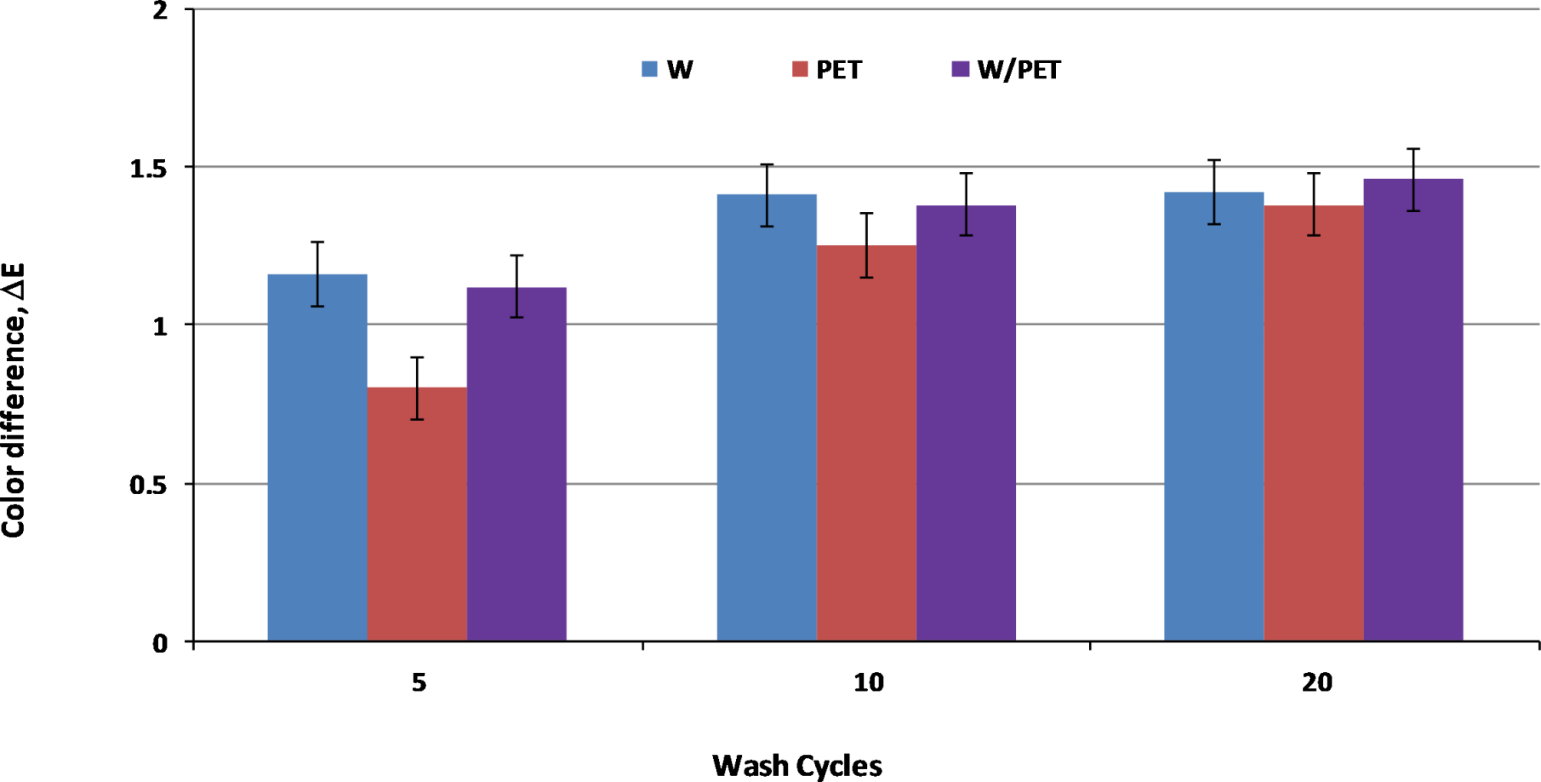
ΔE values of the SC-CO_2_ dyed W, PET and W/PET blend fabrics at different washing cycles.

**Fig. 16:**
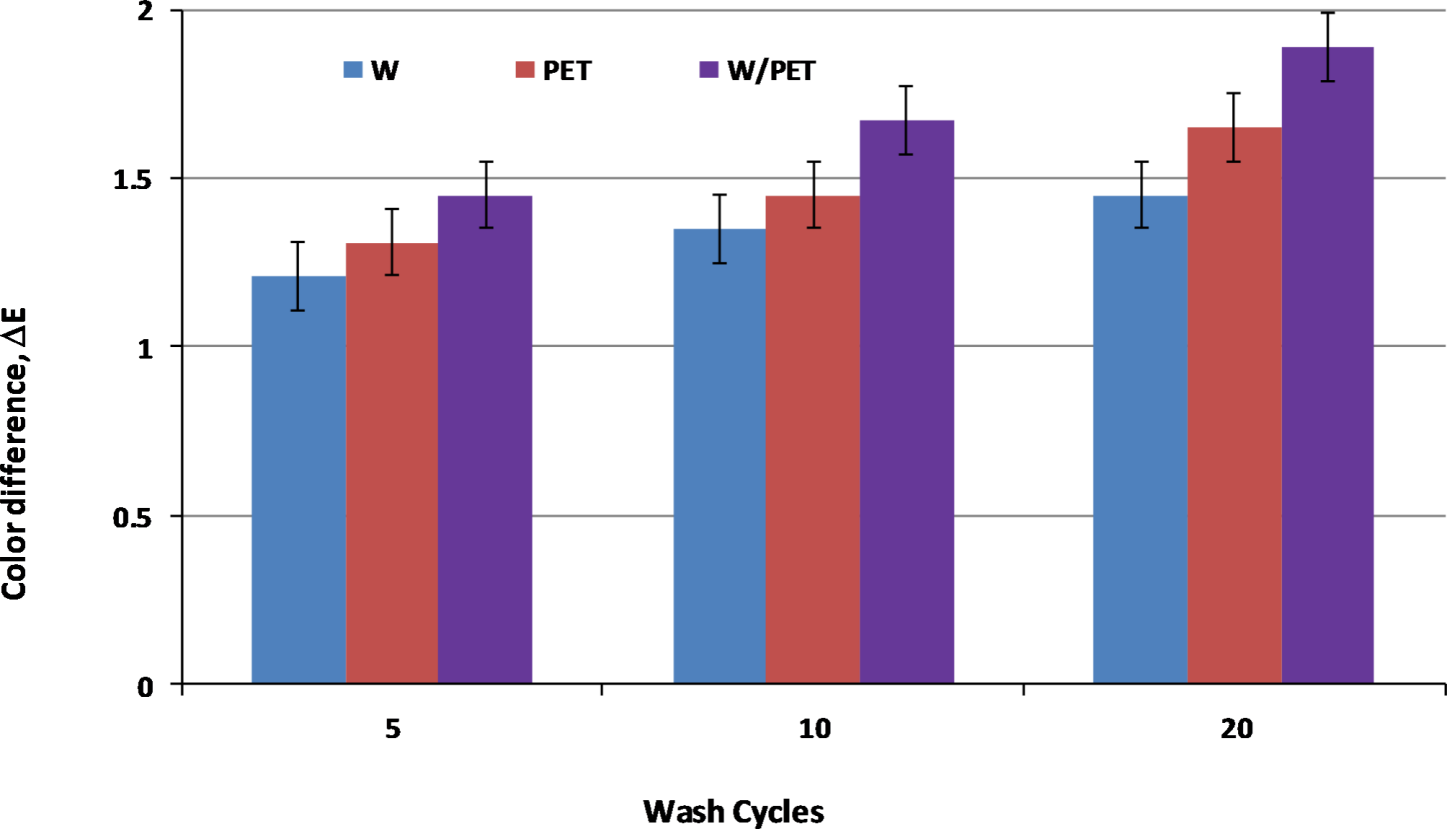
ΔE values of the aqueous dyed W, PET and W/PET blend fabrics at different washing cycles.

Importantly, successive washing cycles (5, 10 and 20 cycles) were carried out for W, PET and W/PET dyed fabrics and ΔE values were evaluated accordingly. The color effects of the dyed fabrics displayed satisfactory ΔE values and both the SC-CO_2_ and aqueous methods showed no significant color loss in the dyed fabrics as shown in Figs. 15 and 16, respectively.

### Fastness properties of SC-CO2 and aqueous dyeings

Table 2 shows data for color fastness to washing, perspiration and light for W, PET, N, W/PET and W/N blended fabrics dyed in SC-CO_2_ medium at (250 bar, 100 °C,2% shade and 1 h) and aqueous medium at (2% shade, 100 °C and 1 h) with VS dye. As listed in Table 2, W, PET, N, W/PET and W/N blended fabrics dyed in SC-CO_2_ and aqueous medium present similar fastness ratings with excellent to very good washing, perspiration and light fastness. Also, both the SC-CO2 and aqueous dyed fabrics showed approximately similar rubbing fastness results with very good to good ratings.

**Table 2:** Fastness properties of W, PET, N, W/PET and W/N blended fabrics dyed in SC-CO_2_ and aqueous media.

Dyed samples	Dyeing method	Rubbing fastness		Washing fastness*			Perspiration fastness*						light
							Acidic			Alkaline			
		Dry	Wet	Alt.	SC	SP	Alt.	SC	SP	Alt.	SC	SP	
W	SC-CO_2_	4–5	4	4–5	4–5	4	4–5	5	4	4–5	4–5	4	6
	Aqueous	4	3–4	4–5	4–5	4	4–5	4–5	4	4–5	4–5	4	6
PET	SC-CO_2_	4–5	4–5	4–5	5	4	4–5	5	4	4–5	4–5	4	5–6
	Aqueous	4–5	4–5	4–5	5	4	4–5	4–5	4	4–5	4–5	4	5–6
N	SC-CO_2_	4	3–4	4–5	4–5	4	4–5	5	4	4–5	4–5	4	6
	Aqueous	4	3–4	4–5	4–5	4	4–5	4–5	4	4–5	4–5	4	6
W/PET	SC-CO_2_	4–5	4	4–5	4–5	4	4–5	5	4	4–5	4–5	4	5–6
	Aqueous	4	3–4	4–5	4–5	4	4–5	5	4	4–5	4–5	4	6
W/N	SC-CO_2_	4	3–4	4–5	4–5	4	4–5	4–5	4	4–5	4–5	4	5–6
	Aqueous	4	3–4	4–5	4–5	4	4–5	4–5	4	4–5	4–5	4	5–6

*Alt color change, *SC* staining on cotton, *SP* staining on polyester.

## Conclusion

A reactive disperse dye containing VS reactive group was successfully designed, prepared and applied to W, PET and N and their W/PET and W/N blended fabrics in SC-CO_2_ medium. Effects of dyeing temperature and pressure on the K/S values of all dyed fabrics were analyzed. The application of VS dye in SC-CO_2_ medium exhibited satisfactory dye uptake performance, color characteristics, leveling properties and color fastness on all fabrics. The detection of higher K/S values on both PET and W/PET fabrics in SC-CO_2_ medium compared with those in aqueous medium can advantageously the application of such type of VS dye structure for SC-CO_2_ dyeing of W/PET blended fabrics on a semi-pilot or industrial scale. These findings can induce an alternative approach to the conventional aqueous dyeing of blend fabrics, which are mainly carried out in two separate dyebaths, resulting in saving time, chemicals, water and dyes. The SC-CO_2_ dyeing also found satisfactory for the type of N fabric products. The nucleophilic addition reaction between the VS reactive group of the dye molecules and nucleophilic sites in W and N fabrics is possible under SC-CO_2_ conditions. The SC-CO_2_ dyeing has a notable influence on a*, b* and C* values of the dyed PET, N and W/PET fabrics and showed that their appearance colors are higher and more saturated than the aqueous dyed samples. All the dyed W, PET and N fabrics and W/PET and W/N blended fabrics show very good levelling properties, indicating that the SC-CO_2_ dyeing method has a substantially better effect on the ∆E values of the dyed samples. The results of successive washing of the dyed W, PET and W/PET blended fabrics showed no significant color change during domestic laundering, even up to 20 cycles. These results can favour this model of VS reactive disperse dye for dyeing PET fabric and its W/PET blend fabrics in SC-CO_2_ medium. Therefore, the effect of the color range of reactive disperse dye structures incorporating different reactive groups and the blend ratios is a crucial point in SC-CO_2_ blends dyeing and further work will merit investigation.

## Data Availability

All data generated or analyzed during this study are included in this published article.
